# Enhancing the upconversion efficiency of NaYF_4_:Yb,Er microparticles for infrared vision applications

**DOI:** 10.1038/s41598-023-35164-x

**Published:** 2023-05-24

**Authors:** Keyvan Khosh Abady, Dinesh Dankhar, Arjun Krishnamoorthi, Peter M. Rentzepis

**Affiliations:** Department of Electrical and Computer Engineering, Texas A&M University, College Station, TX 77843 USA

**Keywords:** Materials for optics, Nonlinear optics

## Abstract

In this study, (NaYF_4_:Yb,Er) microparticles dispersed in water and ethanol, were used to generate 540 nm visible light from 980 nm infrared light by means of a nonlinear stepwise two-photon process. IR-reflecting mirrors placed on four sides of the cuvette that contained the microparticles increased the intensity of the upconverted 540 nm light by a factor of three. We also designed and constructed microparticle-coated lenses that can be used as eyeglasses, making it possible to see rather intense infrared light images that are converted to visible.

## Introduction

Rare earth ions, such as Er^3+^ doped upconverting particles (UCPs), have the unique property of sequentially absorbing two or more low-energy IR photons and emitting a single higher energy, visible photon via a nonlinear stepwise two-photon process^1–4^. This process may be used for a large number of applications, including super-resolution microscopy^5^, enhancing solar cell efficiency^6–8^, detection of latent fingerprints^9,10^, optogenetics^11–13^, high-resolution bioimaging^14–16^, photodynamic therapy^17,18^ and sensing^19,20^.

UCPs are also considered promising alternatives for conventional luminophores such as organic dyes and semiconductor quantum dots^21,22^, because they possess unique optical properties such as a long lifetime of the intermediate energy levels^23–25^, high photostability^3,26^, low chemical toxicity^27^, multiple-peak spectral patterns^3^, lack of photo-blinking even at the millisecond and second-time scales^28^, and absence of photobleaching even after hours of continuous excitation^29,30^. In addition, the scattering of the near-infrared excitation light in many dispersing media such as air, water and biomedical tissues is significantly reduced in comparison to UV and visible light scattering^31–33^, which makes it highly suitable for many applications, including aerospace and biomedicine^6,29,34^.

In our earlier studies, we have shown that these upconverting particles can be utilized to induce infrared vision in humans by converting bright infrared light to visible light^35^. Moreover, it has been shown that the injection of rare-earth-doped upconversion nanoparticles into the eyes of mice enables them to see in the near-infrared light^36^. However, one major limitation regarding such uses of UCPs is that their upconversion quantum yield is typically less than 1% at room temperature^37,38^, due to the small excitation cross-section of the lanthanide doped materials (e.g., Nd^3+^, Ho^3+^, Er^3+^, Tm^3+^, Yb^3+^)^34,35,38^. Therefore, enhancing the upconversion luminescence efficiency is one of the major challenges encountered in this field. To that effect, various approaches have been proposed^21,34,39–41^.

This paper describes a new technique for increasing the interaction of IR light with microparticles and thereby enhancing the intensity of the upconverted visible light by a factor of three.

The luminescence of UCP is dependent upon several factors, including the size and shape of the host matrix and their dopants, the concentration of the sensitizers and activators in the host matrix, the excitation power, and the concentration of the UCPs in the dispersion medium.

The size of the particles is very important regarding the emission efficiency, which influences the efficiency of upconverting luminescence intensity, owing to the fact that particle size determines the surface-to-volume ratio^29^.

By increasing the size of the host crystal, the surface quenching effect will be decreased owing to the low surface-to-volume ratio. This will result in increasing the lifetime of the intermediate quantum energy state, which increases the quantum yield of the emitted photons^34^. However, the upconverted luminescence of the UCPs decreases significantly as the crystal diameter decreases for a given laser excitation power density^21,42^. It has been shown that for a power density of 20 W/cm^2^, the quantum efficiency of micro-sized NaYF_4_:Yb,Er particles is 10.2%, whereas the quantum efficiency of the nano-sized particles is 0.32%^43^. This is the reason that we chose to work with micro-sized particles (1–5 microns in diameter).

The crystal structure of the upconverting particles is known to have a strong influence on the upconversion efficiency. To that effect, it has been shown that hexagonal *β*NaYF_4_ crystals, activated by Yb^3+^ and Er^3+^, are an order of magnitude more efficient than *α*NaYF_4_ crystals^35,44,45^.

A high dopant concentration of sensitizer ions (e.g., Yb^3+^, Nd^3+^, Er^3+^, Ho^3+^) is known to enhance the luminescence intensity of the UCPs. However, an oversaturation of sensitizer concentration will result in decreased luminescence intensity^29,34^. The microstructure of a single UCP crystal usually contains thousands of photon sensitizers and hundreds of photon activators (e.g., Er^3+^, Tm^3+^ or Ho^3+^)^25,29^. Theoretically, it is expected that by increasing the concentration of the sensitizers and activators in the UCP crystal, one may expect that the intensity and efficiency of the upconversion luminescence to increase^46,47^. However, it has been shown that above a certain concentration, a further increase in dopant concentration may lead to luminescence quenching owing to saturation effects^11,47^.

Our data also show that as the concentration of the UCPs [NaYF_4_:Yb,Er] increases in the dispersion medium, the intensity of the upconverted light, as expected, increases linearly^35^. To increase the intensity of the upconverted green light, while the pumping intensity at 980 nm remains the same, we attached 980 nm reflecting mirrors on the sides of the cuvette that contains the upconverting microparticles. For these experiments, we used UCPs which consist of Sodium Yttrium tetra Fluoride (NaYF_4_) crystalline host matrix doped with Erbium (Er^3+^) and Ytterbium (Yb^3+^) lanthanide ions. The Er^3+^ ions act as emitters, while the Yb^3+^ ions are the sensitizers. Upon excitation with a 980 nm laser light, a two-photon stepwise process is initiated, resulting in the absorption of 980 nm light and after a two-photon stepwise absorption, 540 nm green light is emitted.

## Materials and methods

The upconverting particles [NaY_0.77_Yb_0.20_Er_0.03_F_4_] which are 1–5 microns in size (with an average of 2 microns), were purchased from Sigma Aldrich and used as received. The structure of the microparticles was confirmed by XRD, as reported in the corresponding datasheet^48^. A magnetic stirrer was used to continuously stir the solution in order to prevent the microparticles from settling down. A Molectron (PM3Q with EPM1000) optical power meter was utilized to measure the power of the 980 nm excitation light, while the upconverted 540 nm light intensity was measured by a Hamamatsu-R928 photomultiplier tube attached to an oscilloscope. A 1 cm glass cuvette that transmits 85% at both the 980 nm excitation and the 540 nm upconverted wavelengths was utilized in all experiments described. The mirrors we employed in this study were 1 cm × 1 cm × 1 mm made of glass coated with Aluminum. These mirrors were placed on four sides of the cuvette, covering an area of 1 cm × 4 cm on each side. To construct the microparticle coated eye lenses, we made a clear solution of equal amounts of polyepoxides and hardener and added immediately the upconverting microparticles to make a 50 mg/ml solution. Then, the mixture was cured for 48 h at room temperature.

We also utilized a 500 mW/cm^2^ CW diode laser emitting at 980 nm as the excitation light source. The emission spectra were recorded by a Shimadzu RF-5301PC spectrofluorophotometer and a USB spectrometer (B&W Tek), while the absorption spectra were recorded by means of Shimadzu UV-160U and Shimadzu-1201 UV–Vis spectrophotometers. The excitation spectra were recorded by means of a Shimadzu RF5000U spectrofluorophotometer.

The absorption cross-section of the NaYF_4_:Yb,Er microparticles was determined by:

1 $$A=-{\mathit{log}}_{10}\frac{I}{{I}_{0}}=C\times \varepsilon \times L$$

where $A$ is the absorbance, ${I}_{0}$ and $I$ are the intensities of the incident and transmitted light, $C$ is the concentration of attenuating species in the solvent, $\varepsilon$ is the molar absorption coefficient (Liter × mol^−1^ × cm^−1^) at the desired wavelength, and *L* is the optical path length (cm). The molar absorption coefficient was calculated from the ratio of $A$ to $C$.

## Results and discussion

### Excitation and emission spectra of NaYF_4_:Yb,Er UCPs

**Figure 1:**
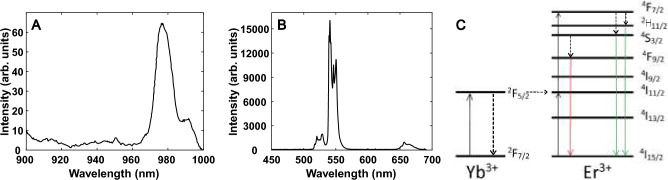
(**A**) Excitation spectrum of the NaYF_4_:Yb,Er micro-crystals and (**B**) the emission spectrum of the NaYF_4_:Yb,Er micro-crystals under 980 nm excitation light. The corresponding energy levels are shown in (**C**).

The excitation spectrum of [NaYF_4_:Yb,Er] when the emission is set at 540 nm is shown in Fig. 1A, where a band with a maximum at 976 nm is clearly visible. The corresponding intense upconverted spectrum emitted by a 980 nm stepwise two-photon process excitation is shown in Fig. 1B.

**Figure 2:**
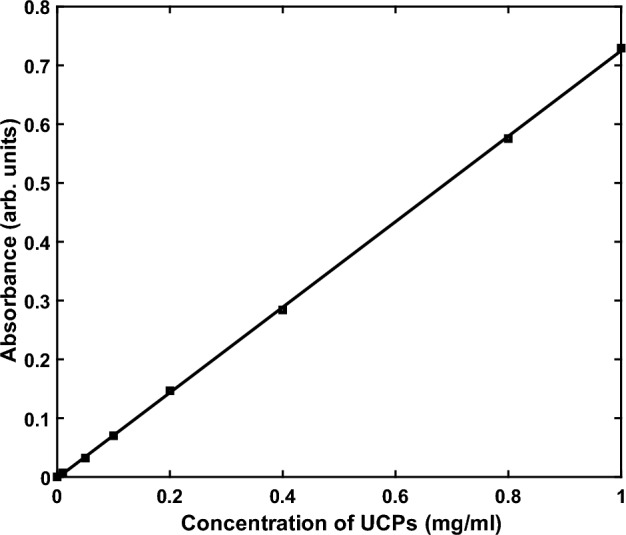
Absorbance of NaYF_4_:Yb,Er at 980 nm vs. concentration in a water solvent.

The 980 nm molar absorption coefficient of NaYF_4_:Yb,Er dispersed in water was determined from the slope of the linear plot shown in Fig. 2 and found to be 150.52 Liter × mol^−1^ × cm^−1^. This value is not dependent on the type of solvent, since we used the same solvent (distilled water) as the reference.

Applying the Beer-Lambert law to the data shown in Fig. 2, we determined that the absorbance depended linearly on the concentration of the UCPs in the solution. As the concentration increased, the absorbance of the 980 nm light in the solution also increased, while the penetration depth decreased. We are investigating whether the dominant mechanism in these values is scattering or absorption in order to determine the accuracy of the calculated extinction coefficient. The intensity of UCPs can be further enhanced by increasing the molar extinction coefficient cross-section at the excitation wavelength, this is possible owing to the fact that the UCPs absorb strongly at 980 nm.

### Effect of the dispersion medium and concentration of UCPs on the intensity of the green upconverted light

**Figure 3:**
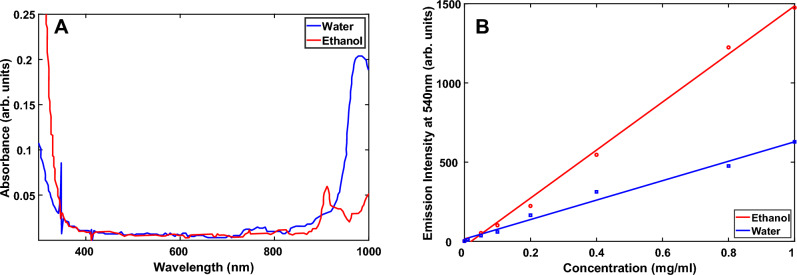
(**A**) Absorption spectra of water and ethanol measured at room temperature and (**B**) upconversion emission intensity measured at 540 nm versus concentration of upconverting particles dispersed in ethanol and water. The excitation power density at 980 nm was 80 mW/cm^2^.

Figure 3A shows the absorption spectra of water and ethanol at room temperature. This figure shows that water has an absorbance of 0.249 at the 980 nm laser pumping wavelength, which suggests that when we excite the sample with 980 nm light, a relatively large percentage of the excitation light intensity is absorbed by the water, and consequently the intensity of the upconverted 540 nm light is proportionally decreased.To avoid this absorption, we repeated this experiment using ethanol as the solvent instead of water. The absorption spectrum of ethanol (Fig. 3A) shows that its absorbance at 980 nm is only 0.068 versus 0.249 for water. Therefore, we used ethanol as the solvent in the experiments presented in this paper.

Figure 3B displays the upconversion emission dependence on the concentration of the upconverting particles dispersed in water and ethanol. For concentrations up to 1 mg/mL, the intensity of the upconverted light is linear with concentration.

### Effect of the mirror induced multi-reflection on the intensity of the green upconverted light under 980 nm excitation

Only a fraction of the infrared light is absorbed by the upconverting particles, whereas the rest of the light is either scattered or transmitted through the sample. The transmitted infrared light was utilized by placing a mirror that reflects this light back into the sample, thereby allowing a second pass through the sample. Ideally, with a single mirror, we can achieve a two-fold enhancement in the upconverted emission intensity. By placing and fixing mirrors on the sides of the cuvette, we can reflect back the scattered infrared light from other directions.

**Figure 4:**
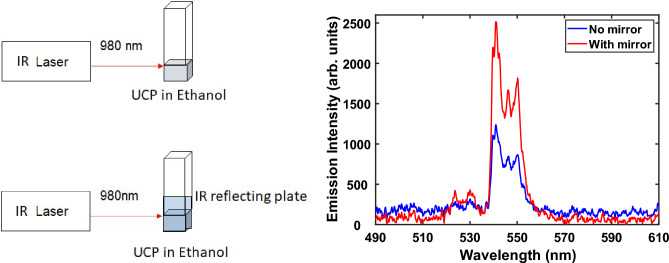
Two times enhancement of upconverted emission with a single mirror.

Figure 4 shows the upconverted emission spectra from a suspension of UCP in ethanol (concentration 0.1 mg/mL) with and without the single back reflecting mirror, under excitation with 980 nm laser light. An enhancement of nearly two times is evident in Fig. 4. When water was used as the dispersion medium, the upconversion emission intensity as well as the enhancement factor were slightly less than 2. This is attributed to the fact that water absorbs part of the 980 nm light, and thereby the reflected light suffers additional attenuation.

**Figure 5:**
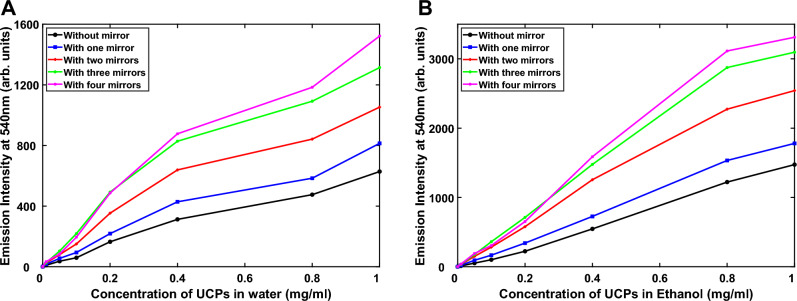
(**A**) Effect on emission intensity at 540 nm by placing one, two, three, and four mirrors on the sides of the cuvette containing UCPs dispersed in water and (**B**) ethanol.

When mirrors are placed on one, two, three and four cuvette sides, the upconverted emission is further enhanced (Fig. 5). The upconverted emission intensities as a function of concentration are shown in Fig. 5A,B for the upconverting particles dispersed in water and ethanol, respectively.

Figure 5A,B illustrate the effect that the mirrors have in enhancing the upconverted visible light intensity, where it is shown that the intensity is increased by nearly a factor of three, for a wide range of concentrations.

Figure 5A,B, as expected, demonstrate the fact that the upconversion emission is more intense when ethanol is used as a dispersion medium compared to water. This is attributed, as mentioned previously, to the low 980 nm absorption by ethanol.

### Effect of the excitation light intensity on upconversion intensity

**Figure 6:**
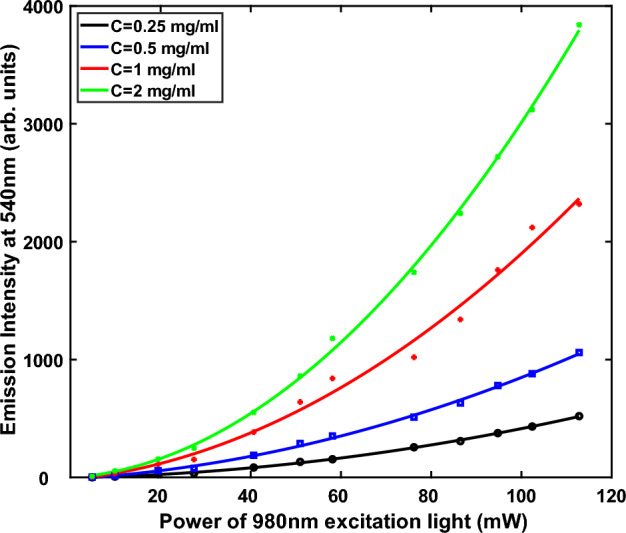
Effect of the concentration of upconverting particles on the slope of the power of excitation light intensity versus the upconverted emission light intensity.

Figure 6 shows the quadratic relationship between the 980 nm input light intensity and the 540 nm output light at various concentrations (0.25, 0.5, 1, 2 mg/ml). The quadratic nature of the curves clearly shows that as the power density increases, the upconversion efficiencies also increase. These data also provide a reason why it is very difficult to achieve upconverted intensities higher than three or four times, even if we place mirrors all around the cuvette. The intensity of the scattered IR light and the IR light after several reflections decreases to an extent (power per unit area), which results in much lower two-photon upconversion efficiencies, even though the remaining IR light is allowed to interact more with the sample after multiple reflections. Figure 6 shows that as the concentration increases, the second derivative of the curve increases, which suggests that by increasing the concentration, the slope of the curve, as expected, also increases.

### Bleaching of Rhodopsin with infrared light

**Figure 7:**
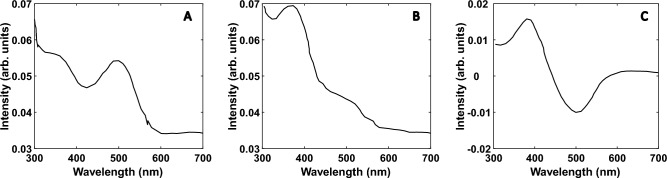
(**A**) Absorption spectra of rhodopsin, before bleaching and (**B**) after bleaching with 980 nm laser light in the presence of upconverting particles and (**C**) the difference absorption spectrum between before and after bleaching spectra.

Another interesting experiment, that we performed, that proved that we converted IR to visible light was the bleaching of bovine rhodopsin (the molecule that induces vision in the eyes), owing to the fact that rhodopsin bleaching is achieved only by visible light (540 nm in this case) and not by 980 nm light. Rhodopsin protein is responsible for low light intensity vision, whose absorption maximum is located at ~ 500 nm. Rhodopsin has zero absorption at 980 nm. A 40 µl solution of rhodopsin showed no bleaching when illuminated with 980 nm, 80 mW laser light for 15 min. In contrast, when we placed upconverting particles in front of the rhodopsin sample and illuminated it with the same intensity of 980 nm IR light, the sample was bleached within milliseconds as it does under visible light (Fig. 7). This showed, very emphatically, that infrared vision could be achieved by using these IR to visible light upconverting particles.

### Broadband, IR and visible vision glasses

**Figure 8:**
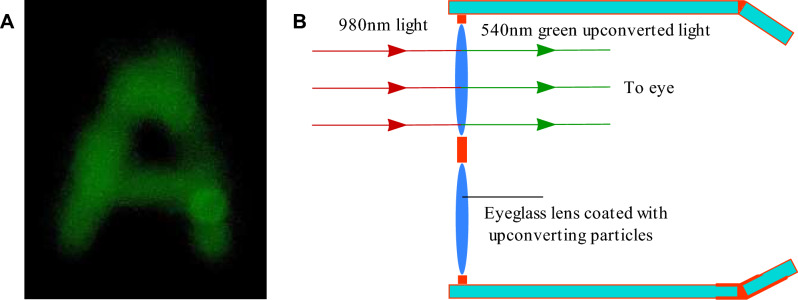
(**A**) Image recorded through the described optical eyeglasses under 980 nm infrared illumination and (**B**) 980 nm to vision glasses fabricated by upconverting micro-particles dispersed in clear resin.

We have designed and constructed optical eyeglasses that upconvert NIR light into visible green light, thus making it possible to detect IR images. These glasses are coated with the previously described particles that upconvert IR to visible light and consist of a filter that transmits visible green light and reflects infrared. When the infrared light image reaches the glass, an image is created by the infrared light on the microparticle-coated glass. The infrared light image is converted to visible (540 nm) image by the upconverting particles, and subsequently it is transmitted and detected by the human eyes situated behind these glasses. Figure 8A displays the image formed on the eyeglass coated with the [NaYF_4_:Yb,Er] upconverting particles under 980 nm infrared illumination. The object was the letter “A” written on a piece of glass and situated in front of the coated eyeglass. The power density of the 980 nm infrared light impinging on this object was ∼40 mW/cm^2^, and the power density of the visible light illumination was ∼1 mW/cm^2^. The picture shown in Fig. 8A was taken by placing a camera behind the coated eyeglass, where the eyes would be located. By placing a near-infrared filter on the backside of the glass, the eyes can be protected from any intense near-infrared light that might pass through, which is expected to enhance the intensity of the green image owing to the fact that the reflected 980 nm light will pass again through the UCPs.

## Conclusion and outlook

We have studied the process of IR to visible light upconversion using rare-earth ion doped micro-particles (NaYF_4_:Yb,Er), which are one of the most efficient sensitizers and activator materials used for up-converting infrared light to visible. In particular, we studied the two-photon stepwise nonlinear process for the conversion of 980 nm IR excitation light to 540 nm visible light. The increase in the intensity of the upconverted green light was studied, also as a function of microparticle concentration and by the placement of reflective mirrors on the sides of the cell that contain the microparticles. This made it possible to increase the intensity of the upconverted 540 nm light several times. In addition, the use of ethanol instead of water as the solvent increased the intensity of 540 nm light significantly.

We, also, were able to utilize upconverting particles to bleach rhodopsin, which is responsible for vision, using 980 nm infrared light, interacting with upconverting particles, which shows that these particles can be used to induce vision in the infrared region. In addition, we designed and constructed thin eyeglass lenses that are coated with the UCPs and can be used to convert NIR images to 540 nm visible light images. We are now studying various nano and micron-sized materials and methods in order to increase severalfold the IR to visible upconversion efficiency.

## Data Availability

The authors confirm that the data supporting the findings of this study are available within the article.
